# Beyond Ocular Symptoms: Chronic Progressive External Ophthalmoplegia With Coexisting Facial Palsy and T-wave Inversion

**DOI:** 10.7759/cureus.116467

**Published:** 2026-09-18

**Authors:** Sangeeta Gupta, Alka Tripathi

**Affiliations:** 1 Physiology, All India Institute of Medical Sciences, Gorakhpur, Gorakhpur, IND; 2 Ophthalmology, All India Institute of Medical Sciences, Gorakhpur, Gorakhpur, IND

**Keywords:** chronic progressive external ophthalmoplegia, cpeo, extraocular, facial, mitochondrial, myopathy, ocular, ptosis

## Abstract

Chronic progressive external ophthalmoplegia (CPEO) exists on a wide phenotypic continuum. The systemic propagation of cellular metabolic impairment frequently precipitates a heterogeneous spectrum of neurogenic, myopathic, and cardiovascular manifestations, clinically designated as CPEO-plus. Beyond the highly susceptible extraocular muscles, other metabolically intensive muscular tissues remain vulnerable to compromised function. Diagnosing CPEO remains challenging because its ocular signs overlap significantly with other conditions. Furthermore, CPEO-plus must be distinguished from Kearns-Sayre syndrome. This case report documents a 43-year-old female patient exhibiting classic ocular manifestations of CPEO, supported by a positive family history and elevated serum lactate levels. The clinical presentation is complicated by atypical features, including mild unilateral facial palsy and signs of cardiac electrical remodeling (T-wave inversion), illustrating the significant phenotypic heterogeneity characteristic of CPEO-plus syndromes.

## Introduction

Chronic progressive external ophthalmoplegia (CPEO) is a rare mitochondrial muscle disease characterized by slowly progressive, painless, and bilateral ptosis along with bilateral ocular immobility that uniquely spares the pupils [1,2]. The worldwide prevalence of CPEO is unknown; however, the incidence of CPEO is one to two per 100,000 [3]. While it frequently manifests as an isolated ocular myopathy, CPEO can present as part of a broader multisystemic disorder known as CPEO-plus, which is even less common. This expanded phenotype involves extraocular tissues with high metabolic demands, such as skeletal muscle, peripheral nerves, renal tubules, and the myocardium [3]. A strong diagnostic challenge arises when patients present with atypical or asymmetric features, which can lead to significant diagnostic delays or misdiagnoses. CPEO stems from mitochondrial DNA (mtDNA) deletions or mutations in nuclear genes controlling mtDNA replication. It shows variable maternal or Mendelian inheritance patterns [2]. Defective oxidative phosphorylation forces cells to rely on glycolysis for adenosine triphosphate (ATP) generation (the Warburg effect). This metabolic shift increases pyruvate and shifts the lactate dehydrogenase equilibrium, raising blood and cerebrospinal fluid lactate levels as a marker of mitochondrial dysfunction [4]. The systemic phenotype may have extraocular involvement, e.g., orbicularis oculi or that of other facial muscles. This can easily mimic standard cranial neuropathies. Orbicularis oculi or generalized facial muscle weakness occurs in CPEO patients [5]. However, it typically presents as a bilateral, symmetrical, and slowly progressive expressionless facies. A presentation resembling unilateral facial palsy is exceedingly rare [6]. This often misleads clinicians toward structural cranial nerve lesions or Bell's palsy. Concurrently, subclinical cardiac involvement is well-documented in mitochondrial myopathies. Cardiac conduction system defects are the most characteristic and critical ECG changes seen in patients with CPEO-plus [7]. ECG changes usually range from minor delays to complete blocks in CPEO-plus. While cardiac involvement in CPEO primarily targets the specialized conduction system, manifesting as PR prolongation or bundle branch blocks, T-wave inversion is a rare CPEO-plus finding. In mitochondrial disease, this strongly points to mitochondrial cardiomyopathy, myocardial strain, or deep structural remodeling [8]. These changes can precede overt cardiomyopathy or life-threatening heart blocks, such as those seen in Kearns-Sayre syndrome (KSS), and should receive more attention in the management of patients with CPEO [7].

This case report details a 43-year-old female presenting with classic ocular signs of CPEO alongside a positive family history and elevated serum lactate. The clinical picture is further nuanced by the concurrent presentation of an atypical focal neurological deficit (mild unilateral facial palsy) and a warning sign of cardiac electrical remodeling (T-wave inversion). It serves to highlight the phenotypic heterogeneity of CPEO-plus syndromes. It underscores the critical importance of a multi-system diagnostic approach, specifically utilizing clinical, biochemical, and electrophysiological profiles to achieve diagnostic clarity when advanced modality findings are absent or uninformative.

## Case presentation

A 43-year-old female presented with drooping of both upper eyelids for the past two years, which was gradually progressive in nature. She complained of occasional fatigue and tingling in the lower half of the body. The family history was positive for her brothers and mother. She had no history of ocular trauma or systemic illness. Six months prior, she was diagnosed with hypothyroidism, for which she received 25 micrograms of levothyroxine sodium until its recent cessation. There was no history of syncopal episodes, palpitations, or known arrhythmias/heart block. No complaints of dysphagia or dysarthria were noted.

On ophthalmological examination, visual acuity was 6/12 in the right eye and 6/18 in the left eye. Pupillary examination showed normal pupils (round, regular, and reactive). Extraocular muscle examination revealed hypofunction of all six extraocular muscles (superior rectus, -2 bilaterally; inferior rectus, -2 bilaterally; lateral rectus, -2 for the right eye and -3 for the left eye; medial rectus, -2 for the right eye and -1 for the left eye; inferior oblique and superior oblique muscles, -2 each bilaterally). Ptosis work-up revealed marginal reflex distance-1 (MRD-1) as -3 for the right eye and -4 for the left eye. MRD-2 was 6 mm bilaterally. Levator palpebrae superioris (LPS) and frontalis overactivation were detected. Palpebral fissure vertical height was 3 mm in the right and 2 mm in the left eye. Bell’s phenomenon was poor in both eyes. The patient was also clinically examined for Marcus Gunn jaw-winking phenomenon, which was ruled out. An ice-pack test was conducted to test for the presence of myasthenia gravis, which was negative bilaterally (no improvement detected). No diplopia or ocular pain was noted. Fundoscopic examination was unremarkable except for the presence of mild temporal pallor in the left eye.

Muscle power was assessed using the Medical Research Council Scale and graded 5/5 globally across major muscle groups of the upper and lower extremities [9]. Deep reflexes were normal. Sensory examination was normal. No ataxia or other abnormal cerebellar signs were noted. No hearing loss was found. Slight asymmetry on the left side of the face was noted during smiling and whistling. Mild lagophthalmos was noted. No other cranial nerve examination findings were remarkable. Cardiovascular system examination was unremarkable; however, her electrocardiogram (ECG) findings revealed characteristic presence of T-wave inversion in V1 to V6 and lead III traces (Figure 1). Echocardiogram findings, however, were normal. The patient's laboratory findings (biochemical, hematological, and electrophysiological) are presented in Table 1. The acetylcholine receptor antibody test (enzyme-linked immunosorbent assay) was negative (0.25 nmol/L) (considered positive when ≥0.5 nmol/L). Serum lactate levels were elevated (3.7 mmol/L) (normal range: 0.7-2.1 mmol/L). Her complete hemogram showed an elevated erythrocyte sedimentation rate (30 mm/hr), elevated total white blood cell count (14.69 x10³ cells /µL), absolute neutrophil count was 8.17 x10³ cells /µL, absolute lymphocyte count was 5.31 x10³ cells /µL, and absolute monocyte count was 1.03 x10³ cells /µL. Total serum creatine phosphokinase (CPK) was within the normal range. Her free triiodothyronine (free T3) and free thyroxine (free T4) levels were within the normal range (chemiluminescence immunoassay). Electroneuronography (ENoG) recorded for the facial muscles revealed relative axonal damage on the left side. There was a 51.4% relative compound muscle action potential (CMAP) amplitude reduction in the left orbicularis oris, i.e., the CMAP was 48.6% of the unaffected side. The left orbicularis oculi muscle showed about a 23% relative reduction, i.e., the CMAP was 77% of the unaffected side (Table 1 and Figure 2). Surface electromyography (EMG) was performed for the bilateral frontalis muscle, which revealed myopathic features, including short duration and low amplitude motor-unit action potentials and an early recruitment pattern in the muscles tested bilaterally. Repetitive nerve stimulation test (RNST) done for orbicularis oculi and abductor digiti minimi did not show decrement in the CMAP response. She was recommended further investigations in the form of genetic testing and muscle biopsy, but the patient and her relatives did not agree to further examinations. The patient was diagnosed as a probable case of CPEO-plus (with highly suggestive mitochondrial DNA inheritance) based on the detailed clinical, electrophysiological, and biochemical investigations. She was prescribed crutch glasses and hydroxypropyl methylcellulose (HPMC) gel. The patient was appropriately instructed to report immediately if she develops cardiovascular symptoms, including palpitations, dyspnea, or chest discomfort. A follow-up visit was planned after four weeks. Frontalis sling (frontalis suspension) surgery was planned for the patient during the visit.

**Figure 1:**
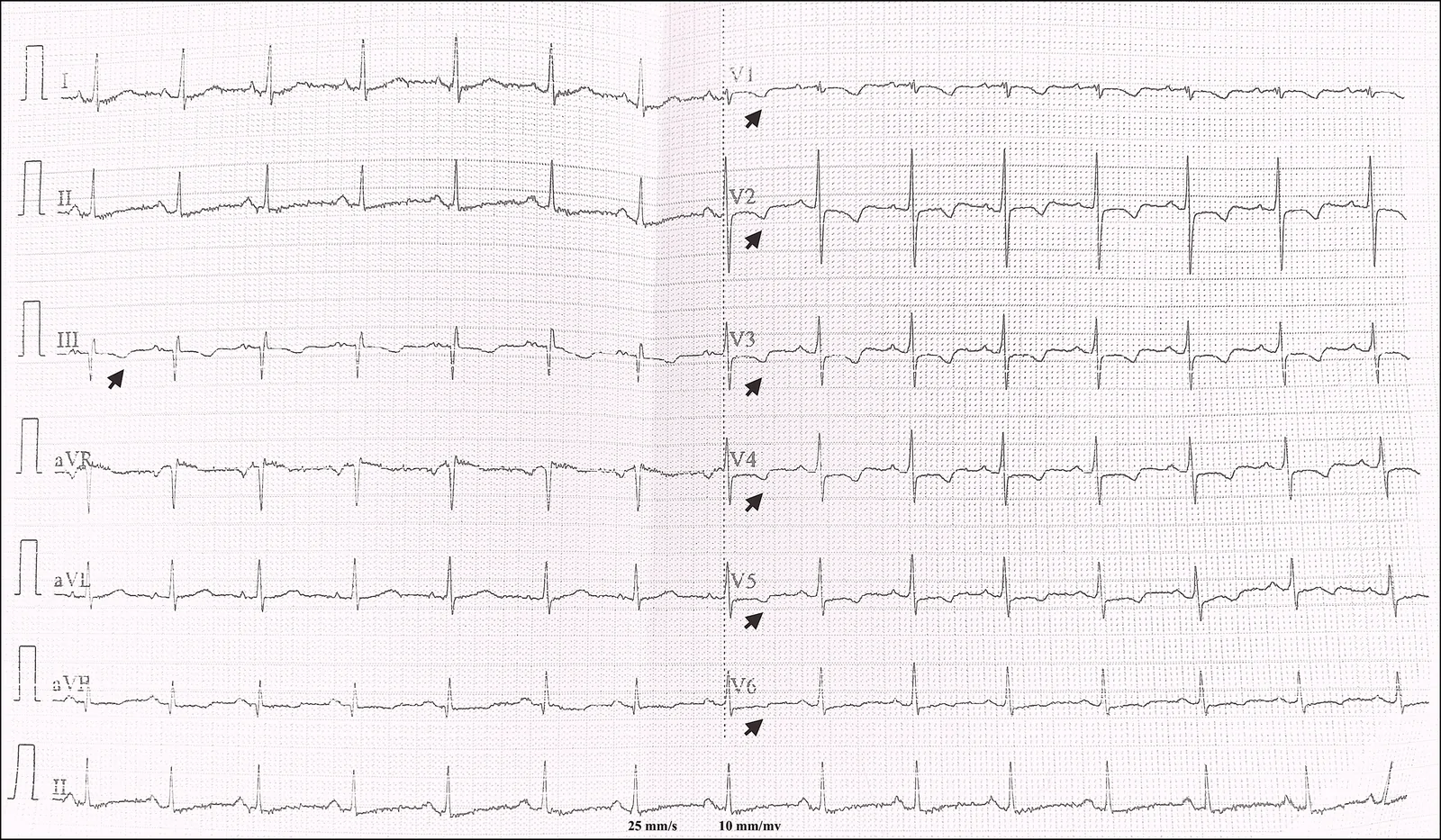
ECG of the patient showing diffuse T-wave inversion in precordial leads (V1-V6) and lead III (black arrows). Heart rate: 88 bpm; PR interval: 156 ms; QT/QTC: 370/450 ms. ECG: electrocardiogram; bpm: beats per minute; ms: milliseconds.

**Table 1:** Laboratory findings. ELISA: enzyme-linked immunosorbent assay; nmol/L: nanomole per liter; mmol/L: millimole per liter; ESR: erythrocyte sedimentation rate; mm/hr: millimeter per hour; µL: microliter; U/L: unit per liter; pg/mL: picogram per milliliter; ng/dL: nanogram per deciliter; ENoG: electroneuronography; CMAP: compound muscle action potential; mv: millivolt.

S. No.	Laboratory investigation	Result	Normal range/cut-off value	
1	Acetylcholine receptor antibody test (ELISA)	0.25 nmol/L	<0.40 nmol/L (negative result)	
2	Serum lactate	3.7 mmol/L	0.7-2.1 mmol/L	
3	ESR	30 mm/hr	0 to 20 mm/hr	
4	Total white blood cell count	14.69 x10³ cells/µL	4 to10 x10³ cells/µL	
5	Absolute neutrophil count	8.17 x10³ cells/µL	2 to 7 x10³ cells/µL	
6	Absolute lymphocyte count	5.31 x10³ cells/µL	1 to 4.8 x10³ cells/µL	
7	Absolute monocyte count	1.03 x10³ cells/µL	0.2 to 0.8 x10³ cells/µL	
8	Total serum creatine phosphokinase (CPK)	60 U/L	30 to 135 U/L	
9	Free triiodothyronine (free T3) (chemiluminescence immunoassay)	3.73 pg/mL	2.0 to 4.4 pg/mL	
10	Free thyroxine (free T4) (chemiluminescence immunoassay)	1.54 ng/dL	0.8 to 1.8 ng/dL	
11	ENoG test	Orbicularis oculi: CMAP amplitude	Left	Right
			2.1 mv	2.7 mv
		Orbicularis oris: CMAP amplitude	Left	Right
			1.7 mv	3.5 mv

**Figure 2:**
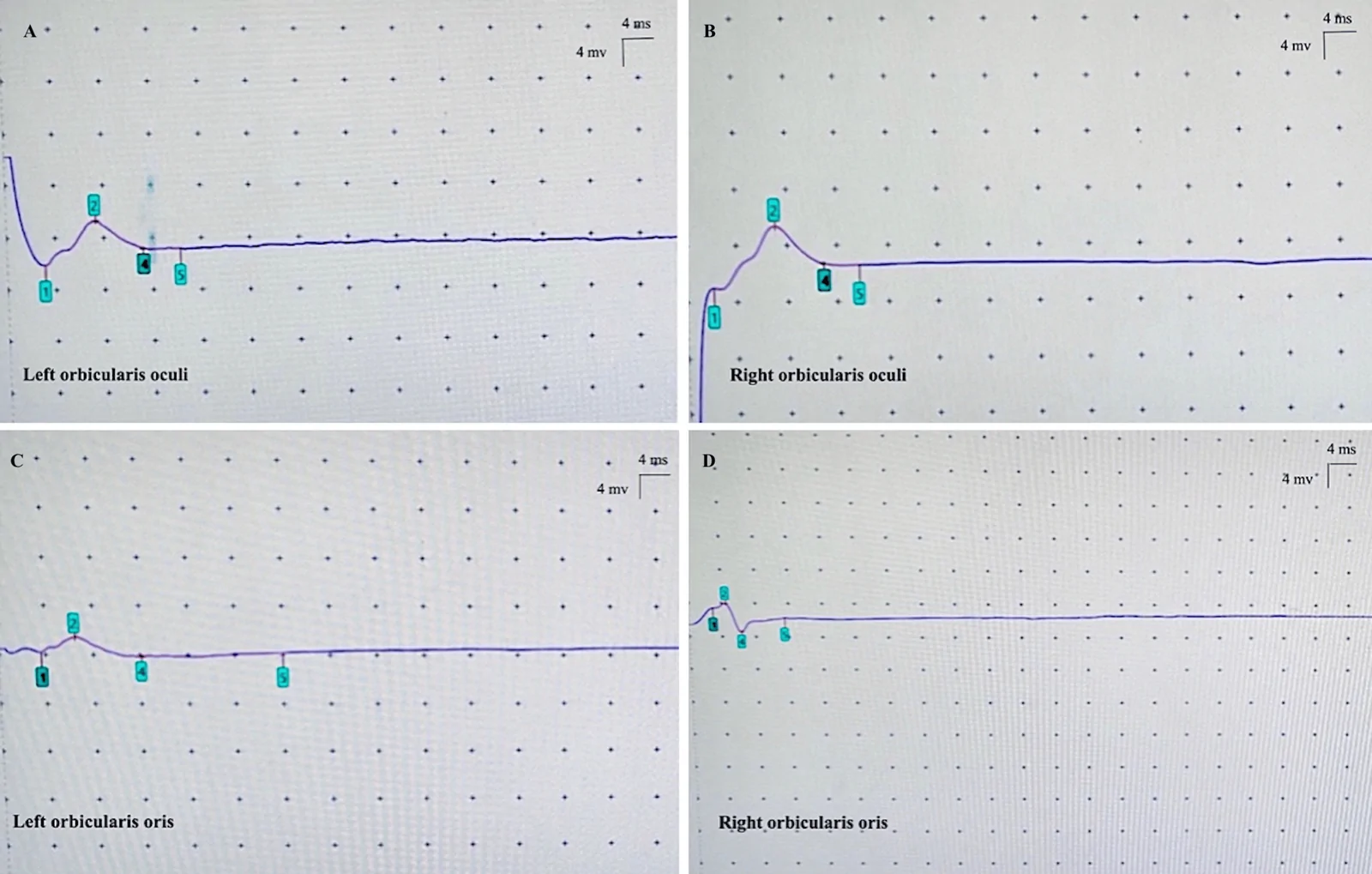
ENoG records for the orbicularis oculi and orbicularis oris muscles in the patient. A relative reduction (left/right) in the left CMAP amplitude (peak-to-peak) of about 23% for the orbicularis oculi (A and B) and 51.4% for the orbicularis oris (C and D) was found. ENoG: electroneuronography; CMAP: compound muscle action potential.

## Discussion

The present report represents a case of CPEO with clinical features beyond ocular involvement. The presence of slowly progressive, painless, and relatively symmetrical bilateral ptosis and ophthalmoparesis, along with pupillary sparing and absence of diplopia, strengthened the clinical diagnosis of CPEO. However, facial muscle weakness and abnormal ECG expanded the clinical spectrum in the present case and rendered the diagnosis in favor of probable CPEO-plus [1]. Systemic phenotype (CPEO-plus) with extraocular involvement commonly leads to orbicularis oculi or generalized facial weakness, which may mimic standard cranial neuropathies. We observed mild facial asymmetry and lagophthalmos on the left side. While classic CPEO presents as bilateral and roughly symmetric ophthalmoparesis, mitochondrial myopathies are increasingly recognized for considerable clinical and phenotypic asymmetry. Focal or homolateral hemifacial weakness and asymmetrical orbicularis oculi involvement can occur as part of the disease spectrum [1]. Incomplete eyelid closure (lagophthalmos) frequently arises in CPEO due to progressive weakness of the orbicularis oculi muscle compounded by structural changes and variable levator/retractor dyskinesia. Also, there was lack of onset of facial weakness as acute and painful in the present case. Moreover, myopathic features in unaffected muscle (frontalis) suggested underlying mitochondrial myopathy rather than a superimposed neuropathy. Raised serum lactate acted as a classic biochemical clue of mitochondrial dysfunction. Moreover, cardiac involvement on ECG pointed toward the possibility of KSS. However, the age of manifestation and absence of pigmentary retinopathy (a core diagnostic hallmark) excluded KSS [10]. Ptosis and ophthalmoparesis often raise the suspicion of ocular myasthenia gravis. Absence of diplopia, no diurnal variation, negative ice-pack test, and negative RNST helped in ruling out the condition.

Elevated serum lactate levels reflect impaired oxidative phosphorylation and a shift toward anaerobic metabolism, serving as a classic biochemical clue of mitochondrial dysfunction [11]. As CPEO is inherently a mitochondrial myopathy, a persistent elevation in blood or cerebrospinal fluid lactate shows that the metabolic defect is not restricted solely to the ocular muscles, but impacts systemic cellular respiration [11]. High or rising lactate often serves as a red flag that the condition may involve more than isolated eye muscle weakness. A normal echocardiogram with diffuse T-wave inversion on ECG in the present case may indicate subclinical involvement. In mitochondrial disorders, electrical and conduction system issues often appear long before any structural changes or overt cardiomyopathy become evident on an ultrasound/echocardiogram [12]. We found normal serum creatine kinase (CK) levels in the patient, which is a usual finding in CPEO/CPEO-plus. Significant muscle fiber breakdown (which causes high CK spikes in inflammatory myopathies or dystrophies) is usually absent in this condition. As these findings argue against active, acute inflammatory myopathies or severe muscle fiber necrosis, the mild elevation in total and absolute counts of white blood cells in the hemogram of the patient may be secondary to an independent, non-muscular inflammatory or infectious trigger.

## Conclusions

The case findings underscore the multisystemic nature of CPEO. It should be borne in mind that the manifestations of the condition may extend beyond isolated ocular involvement. Although the patient exhibited hallmark bilateral ptosis, the concurrent manifestation of facial palsy, elevated serum lactate concentrations, and electrocardiographic abnormalities substantiates a diagnosis in favor of CPEO-plus and corroborates a systemic mitochondrial etiology, independent of overt structural cardiomyopathy or myopathy. This case also demonstrates the role of amalgamating clinical, biochemical, and electrophysiological profiles to attain diagnostic precision when advanced investigative modalities are unavailable.
